# Kaempferol stimulates gene expression of low-density lipoprotein receptor through activation of Sp1 in cultured hepatocytes

**DOI:** 10.1038/srep24940

**Published:** 2016-04-25

**Authors:** Ayasa Ochiai, Shingo Miyata, Masamori Iwase, Makoto Shimizu, Jun Inoue, Ryuichiro Sato

**Affiliations:** 1 Department of Applied Biological Chemistry, Graduate School of Agricultural and Life Sciences, The University of Tokyo, Tokyo 113-8657, Japan

## Abstract

A high level of plasma low-density lipoprotein (LDL) cholesterol is considered a risk factor for atherosclerosis. Because the hepatic LDL receptor (LDLR) is essential for clearing plasma LDL cholesterol, activation of LDLR is a promising therapeutic target for patients with atherosclerotic disease. Here we demonstrated how the flavonoid kaempferol stimulated the gene expression and activity of LDLR in HepG2 cells. The kaempferol-mediated stimulation of LDLR gene expression was completely inhibited by knockdown of Sp1 gene expression. Treatment of HepG2 cells with kaempferol stimulated the recruitment of Sp1 to the promoter region of the LDLR gene, as well as the phosphorylation of Sp1 on Thr-453 and Thr-739. Moreover, these kaempferol-mediated processes were inhibited in the presence of U0126, an ERK pathway inhibitor. These results suggest that kaempferol may increase the activity of Sp1 through stimulation of Sp1 phosphorylation by ERK1/2 and subsequent induction of LDLR expression and activity.

Atherosclerotic cardiovascular disease is a major cause of morbidity and mortality worldwide. Elevated plasma low-density lipoprotein (LDL) cholesterol level is a prominent risk factor for atherosclerosis and coronary heart disease^1^. The LDL receptor (LDLR) was identified as a causative gene for familial hypercholesterolemia^2^, and the liver is a major organ that metabolizes plasma LDL through LDLR-mediated endocytosis^3^. Therefore, up-regulation of LDLR expression in the liver suppresses plasma LDL cholesterol levels and improves atherogenic lipid profiles.

Intracellular cholesterol levels tightly regulate LDLR gene expression through alteration of sterol regulatory element-binding protein (SREBP) activity. When intracellular cholesterol levels are low, proteolytic processing activates SREBP to stimulate the expression of target genes, such as the LDLR and cholesterogenic genes^4^. Sp1 is a zinc-finger type of transcription factor that is ubiquitously expressed and is involved in the regulation of a large number of genes^5^. The promoter region of the LDLR gene contains both SREBP- and Sp1-binding elements, and interaction between SREBP and Sp1 is required for normal sterol regulation of the LDLR promoter^6^.

Kaempferol is one of the flavonols that are abundant in fruits and vegetables^7^,^8^. It has several pharmacologic activities against cancer, atherosclerosis, and hyperlipidemia^9^,^10^,^11^. The molecular mechanism of these activities of kaempferol has been extensively investigated and is becoming gradually apparent^10^,^11^.

To identify compounds that increase the expression of LDLR, we previously established a stable cell line that expressed a luciferase reporter gene under the control of the LDLR promoter^12^. Using this cell line, we examined approximately 100 food components and found several compounds that increased LDLR expression. Using this cell line, in the present study, we have identified for the first time that the flavonoid kaempferol is an inducer of LDLR gene expression. Kaempferol stimulated the activity of Sp1 through its phosphorylation on Thr-453 and Thr-739 by ERK1/2, which increased DNA binding of Sp1 to the promoter region of the LDLR gene.

## Results

### Kaempferol increases LDLR expression and activity

**Figure 1:**
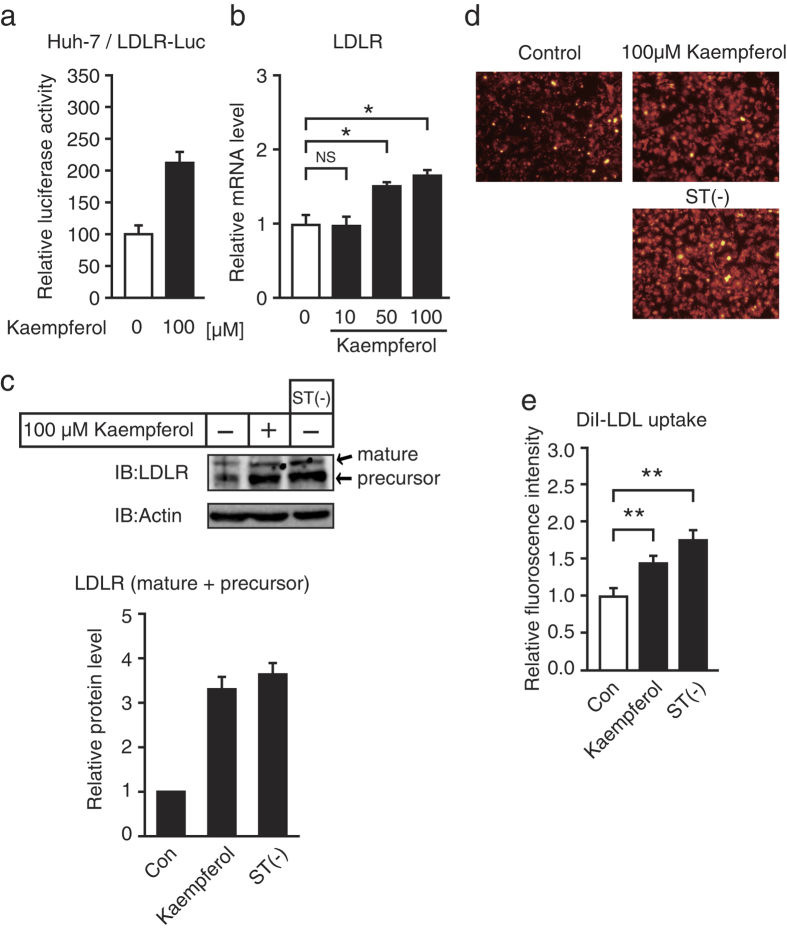
Kaempferol increases the expression and activity of LDLR. (**a**) Luciferase activity in the presence of kaempferol (100 μM). Luciferase activity in the presence of a vehicle is represented as 100. (**b,c**) HepG2 cells were cultured with different concentrations of kaempferol or under sterol-depleted conditions (medium B) for 24 h; total RNA and whole cell extracts were isolated. (**b**) Real-time PCR analysis was performed; mRNA levels were normalized to those of GAPDH mRNA and were expressed relative to those in vehicle (DMSO)-treated controls. (**c**) Whole cell extracts were subjected to SDS–PAGE and immunoblotting (IB) with anti-LDLR or anti-β-actin antibodies. The signals (n = 3) were quantified and normalized by β-actin signals, and the signals of the control group were represented as one. (**d,e**) HepG2 cells were cultured with kaempferol (100 μM) or in medium B under sterol-depleted conditions for 24 h followed by culture in a medium supplemented with 10 μg/ml of DiI-labeled LDL for the last 5 h. (**d**) The cells were then examined using fluorescence microscopy. (**f**) Relative fluorescence levels were normalized to total cellular protein contents. All data are expressed as mean ± SEM, *n* = 3. *P < 0.05, **P < 0.01. NS = not significant.

We have previously established a stable cell line that expressed the luciferase reporter gene under the control of the LDLR promoter region from −595 to +36. In the present study, we used this stable cell line and found that kaempferol stimulated the promoter activity of the LDLR gene (Fig. 1a). Treatment with kaempferol for 24 h caused an increase in the LDLR mRNA level in hepatoma HepG2 cells (Fig. 1b). LDLR protein was synthesized as a precursor with an apparent molecular mass of 120 kDa (Fig. 1c); it was then post-translationally modified into the mature form with an apparent molecular mass of 160 kDa (Fig. 1c). The expression level of LDLR has been known to increase when cells were cultured under sterol-depleted conditions. As shown in Fig. 1c–e, the mature and precursor LDLR proteins as well as fluorescent-labeled LDL (DiI-LDL) uptake for 5 h increased when the cells were cultured for 24 h under sterol-depleted conditions. Treatment with kaempferol for 24 h also increased the LDLR proteins (Fig. 1c) and LDL uptake (Fig. 1d,e) in HepG2 cells. These results indicate that kaempferol stimulates LDLR gene expression and led to increased LDL uptake.

### Kaempferol exhibits a low cytotoxicity to HepG2 cells

**Figure 2:**
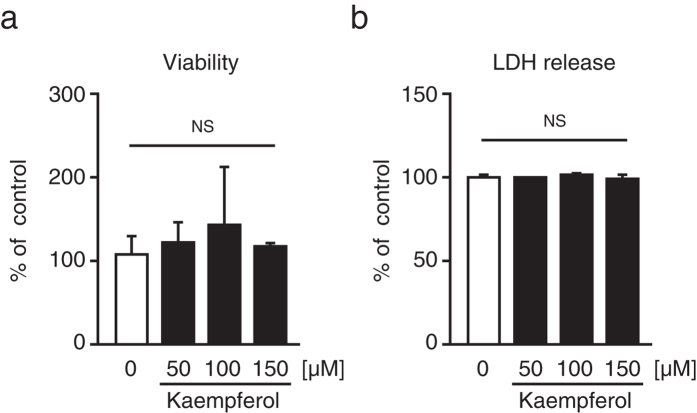
Effects of kaempferol on cell viability and LDH release from HepG2 cells. HepG2 cells were treated with the indicated concentrations of kaempferol for 24 h, followed by MTT (**a**) and LDH (**b**) assays. All data are expressed as mean ± SEM, *n* = 3. NS = not significant.

We investigated the cytotoxic effects of different kaempferol concentrations (50 μM, 100 μM, and 150 μM) on HepG2 cells via 3-(4,5-dimethylthiazol-2-yl)-2, 5-diphenyltetrazolium bromide (MTT) and lactate dehydrogenase (LDH) analyses. As shown in Fig. 2a,b, treatment with kaempferol for 24 h did not affect the cell viability and the release of LDH from HepG2 cells, indicating that kaempferol exhibited a low cytotoxicity to the cells. Therefore, 100 μM kaempferol was used for further experiments.

### The transcription factor Sp1 is required for kaempferol-mediated stimulation of LDLR gene expression

**Figure 3:**
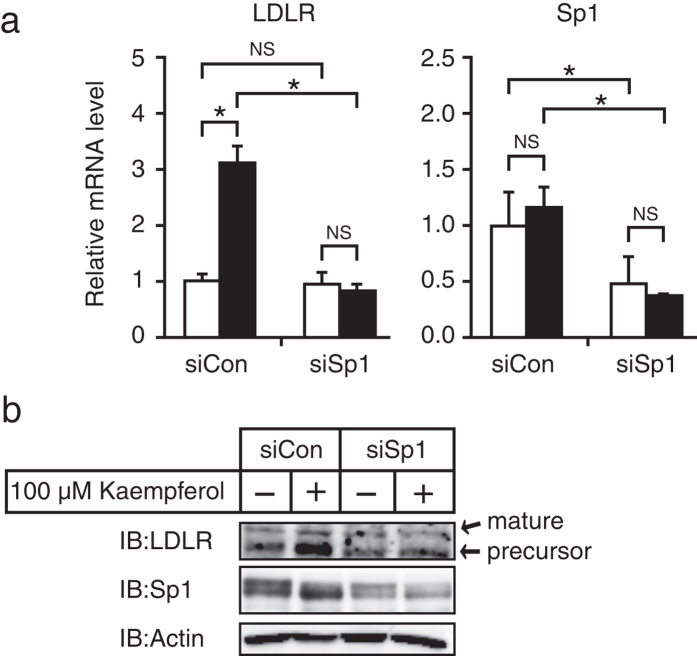
Involvement of Sp1 in kaempferol-mediated stimulation of LDLR gene expression. (**a,b**) HepG2 cells were transfected with 40 pmol of control siRNA (siCon) or siRNA targeting Sp1. Cells were then cultured in medium A for 48 h and were treated with kaempferol (100 μM) for 24 h. (**a**) Total RNA was isolated and real-time PCR analysis was performed. Target mRNA expression levels were normalized to those of GAPDH mRNA and were presented relative to those in vehicle (DMSO)-treated controls. (**b**) Whole cell extracts were subjected to SDS–PAGE and immunoblotting (IB) with anti-LDLR, anti-Sp1, or anti-β-actin antibodies. All data are expressed as mean ± SEM, *n* = 3. *P < 0.05. NS = not significant.

Because it was known that Sp1 regulates the promoter activity of the LDLR gene, we investigated the contribution of Sp1 to kaempferol-mediated stimulation of LDLR gene expression. Transfection of Sp1 siRNA resulted in a 50% decrease in Sp1 mRNA and protein levels and completely inhibited the kaempferol-mediated stimulation of LDLR mRNA and protein expression (Fig. 3a,b). These results indicate that Sp1 is required for kaempferol-mediated stimulation of LDLR gene expression.

### Kaempferol stimulates the recruitment of Sp1 to the promoter region of the LDLR gene

**Figure 4:**
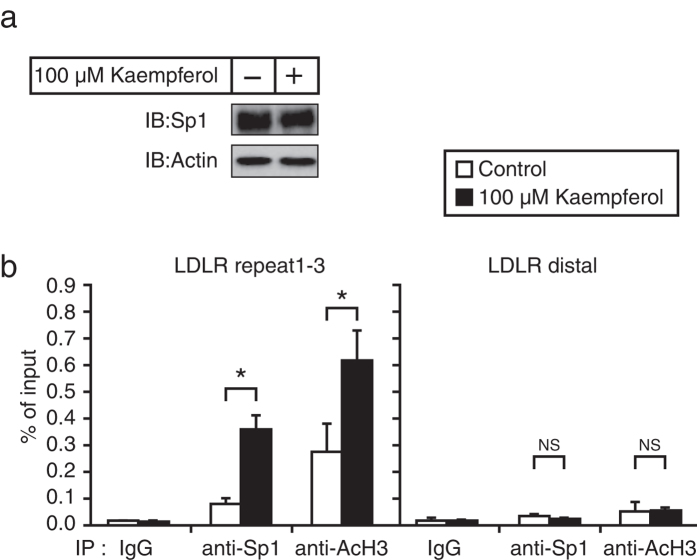
Kaempferol stimulates DNA binding of Sp1 in HepG2 cells. (**a**) HepG2 cells were cultured with kaempferol (100 μM) for 24 h. Whole cell extracts were subjected to SDS–PAGE and immunoblotting (IB) with anti-Sp1 or anti-β-actin antibodies. (**b**) HepG2 cells were cultured with kaempferol (100 μM) for 24 h and processed for ChIP analyses, as described in the Methods section. After immunoprecipitation (IP) with anti-Sp1, anti-acetyl-histone H3, or control IgG, real-time PCR analysis was performed with a primer set covering the Sp1-binding region (repeat1–3) or distal region in the human LDLR promoter. Bound DNA was normalized to the input. All data are expressed as mean ± SEM, n = 3. *P < 0.05. NS = not significant.

Next, we investigated whether kaempferol stimulates the function of Sp1. Kaempferol treatment did not increase the Sp1 mRNA level but slightly reduced the Sp1 protein level (Figs 3a,b and 4a). Following this, we employed a chromatin immunoprecipitation (ChIP) assay to determine whether kaempferol treatment influenced DNA binding of Sp1. The ChIP assay revealed that Sp1 was able to bind to the LDLR promoter region that contained the Sp1-binding elements (repeat 1–3). Kaempferol treatment stimulated this binding and accelerated the recruitment of acetyl-histone H3 to the promoter region of LDLR (Fig. 4b). It should be noted that Sp1 and acetyl-histone H3 did not bind to the distal region of the LDLR promoter, and kaempferol did not influence these processes (Fig. 4b). The distal region of the LDLR promoter is used as a negative control because this region does not contain an Sp1-binding element. These results indicate that kaempferol treatment stimulates the recruitment of Sp1 to the promoter region of LDLR gene.

### Kaempferol stimulates the phosphorylation of Sp1 by ERK1/2

**Figure 5:**
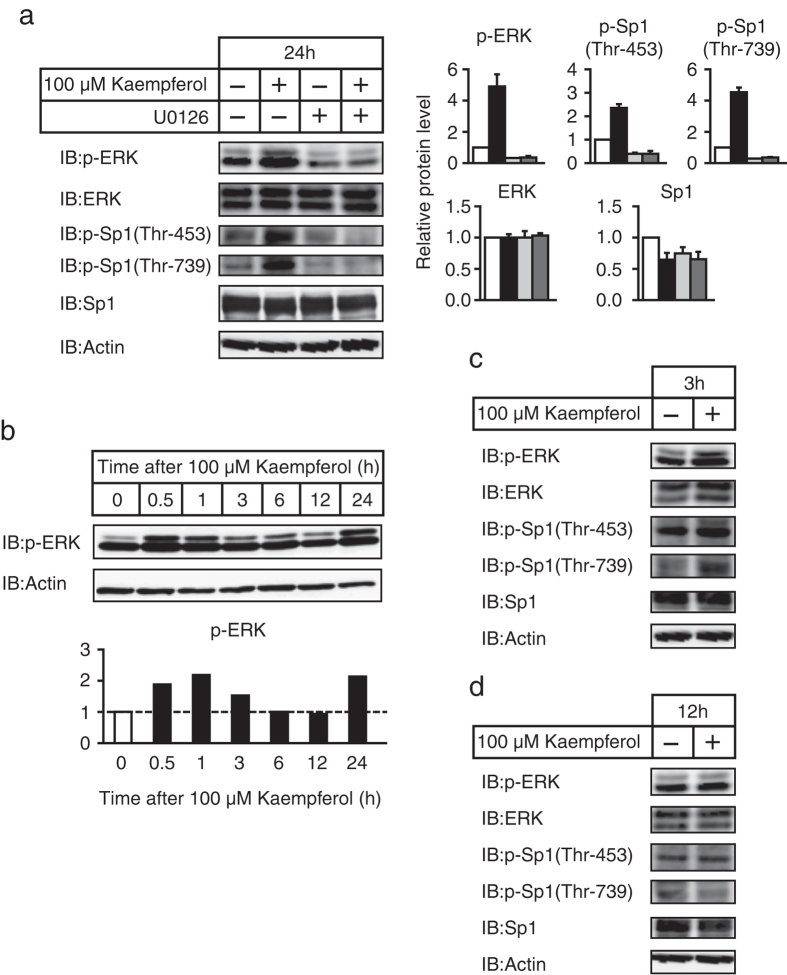
Effect of kaempferol on the ERK pathway and Sp1phosphorylation in HepG2 cells. HepG2 cells were treated with kaempferol (100 μM) for the indicated times in the presence or absence of U0126 (10 μM). Whole cell extracts were subjected to SDS–PAGE and immunoblotting (IB) with anti-p-ERK, anti-ERK, anti-p-Sp1(Thr-453), anti-p-Sp1(Thr-739), anti-Sp1, or anti-β-actin antibodies. (**a**) The signals (n = 3) were quantified and normalized by β-actin signals, and the signals of the control group were represented as one. (**b**) The signals were quantified and normalized by β-actin signals, and the signals of the control group were represented as one. Similar results were obtained in two separate experiments. (**c**,**d**) Similar results were obtained in two separate experiments.

Previous studies have shown that Sp1 on Thr-453 and Thr-739 were directly phosphorylated by ERK1/2 to increase the DNA binding of Sp1^13^. In addition, it has been reported that kaempferol treatment activated the ERK pathway in several types of cells^14^,^15^. This line of evidence led us to consider a possible role of the ERK pathway in kaempferol-mediated stimulation of Sp1 DNA binding. Although stimulation of ERK phosphorylation is commonly analyzed within minutes after treatment, we tested the ability of kaempferol to increase ERK1/2 phosphorylation using samples that were treated for 24 h for consistency with the ChIP assay performed (Fig. 4b). As shown in Fig. 5a, kaempferol treatment increased the phosphorylation of ERK1/2 and was accompanied by an increase in Sp1 phosphorylation on Thr-453 and Thr-739. To determine whether activation of the ERK pathway is responsible for Sp1 phosphorylation in kaempferol-treated cells, we performed experiments using U0126, a specific MEK inhibitor of the ERK pathway. As shown in Fig. 5a, kaempferol-mediated induction of ERK1/2 phosphorylation was stopped after addition of U0126. Importantly, the kaempferol-mediated induction of Sp1 phosphorylation on Thr-453 and Thr-739 was completely inhibited in the presence of U0126, suggesting the involvement of the ERK pathway in kaempferol-mediated induction of Sp1 phosphorylation. These results imply that kaempferol treatment stimulates the DNA binding of Sp1 through phosphorylation of Sp1 by ERK1/2. Further, we examined the time-dependent ERK1/2 phosphorylation by kaempferol. As shown in Fig. 5b, kaempferol increased ERK1/2 phosphorylation at an early time between 0.5 h and 3 h after treatment. This induction disappeared at 6–12 h after treatment, and ERK1/2 phosphorylation increased again at 24 h after treatment. Next, we determined the effect of kaempferol on ERK1/2 and Sp1 phosphorylation after 3 h and 12 h of treatment. As shown in Fig. 5c,d, treatment with kaempferol for 3 h increased Sp1 phosphorylation, especially on Thr-739, whereas treatment with kaempferol for 12 h did not affect it.

### Activation of the ERK pathway is required for stimulation of Sp1 DNA binding and LDLR gene expression by kaempferol

**Figure 6:**
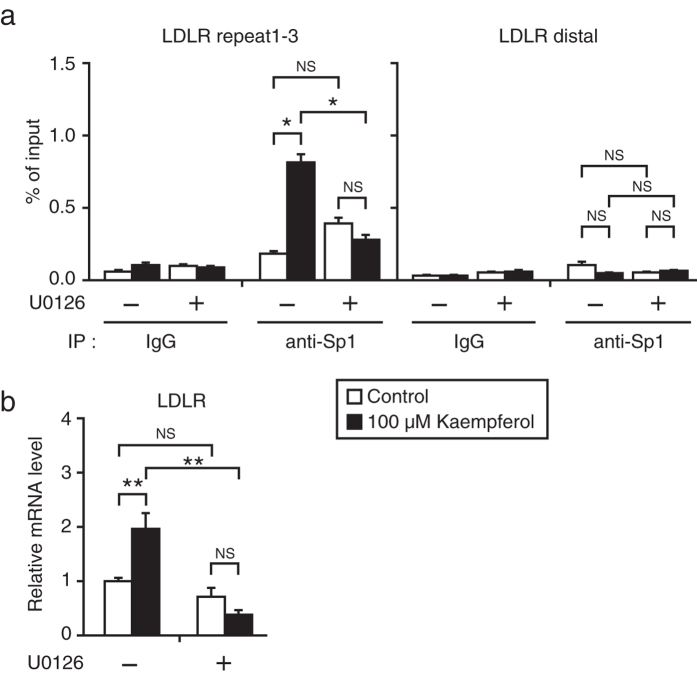
Involvement of the ERK pathway in kaempferol-mediated stimulation of LDLR gene expression. (**a**) HepG2 cells were cultured with kaempferol (100 μM) for 24 h, with and without U0126 (10 μM), and were processed for ChIP analyses, as described in the Methods section. After immunoprecipitation (IP) with anti-Sp1 or control IgG, real-time PCR analysis was performed with a primer set covering the Sp1-binding region (repeat1–3) or distal region in the human LDLR promoter. (**b**) HepG2 cells were treated with kaempferol (100 μM) for 24 h, with or without U0126 (10 μM). Total RNA was isolated and real-time PCR analysis was performed. mRNA levels were normalized to those of GAPDH mRNA and were expressed relative to those in vehicle (DMSO)-treated controls. All data are expressed as mean ± SEM, *n* = 3. *P < 0.05, **P < 0.01. NS = not significant.

Next, we examined whether activation of the ERK pathway contributed to the stimulation of Sp1 DNA binding and LDLR gene expression by kaempferol. The ChIP assay revealed that kaempferol-mediated stimulation of Sp1 DNA binding was inhibited in the presence of U0126 (Fig. 6a). Consistent with this observation, kaempferol could not stimulate LDLR gene expression in the presence of U0126 (Fig. 6b). These results indicate that activation of the ERK pathway is required for kaempferol-mediated stimulation of LDLR gene expression.

### Effects of other flavonoids on LDLR gene expression and ERK pathway

**Figure 7:**
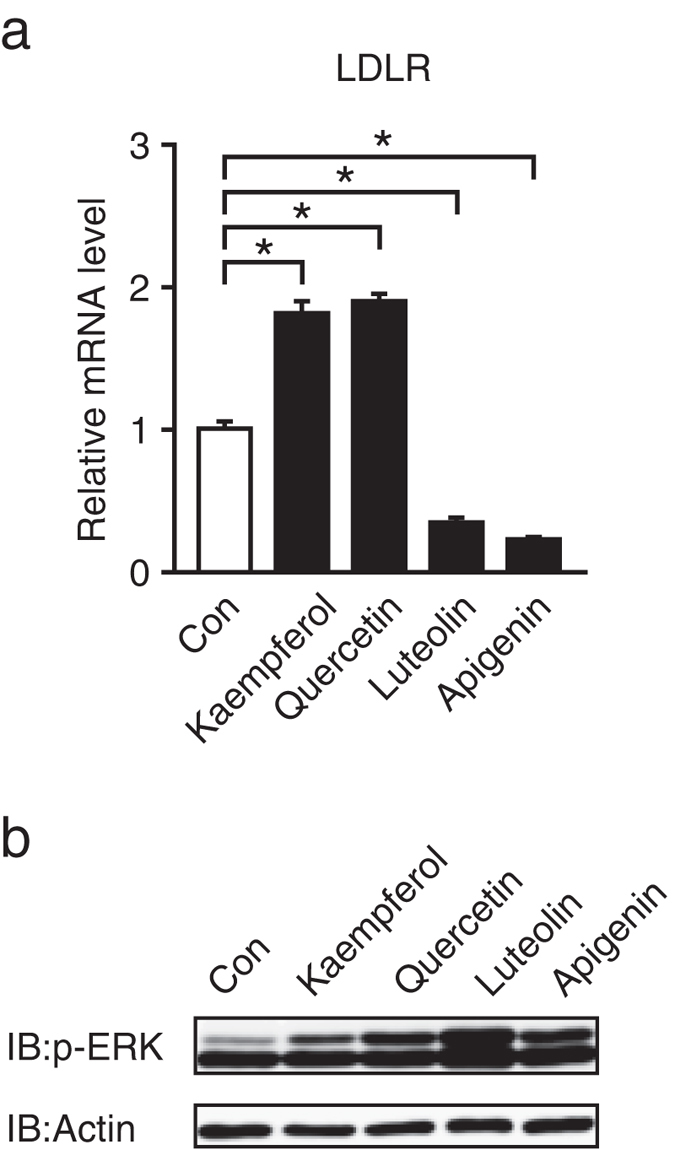
Effects of other flavonoids on LDLR gene expression and ERK pathway. HepG2 cells were cultured with the indicated flavonoids (100 μM) for 24 h, and total RNA and whole cell extracts were isolated. (**a**) Real-time PCR analysis was performed. mRNA levels were normalized to those of GAPDH mRNA and were expressed relative to those in the vehicle (DMSO)-treated controls. All data are expressed as mean ± SEM, *n* = 3. *P < 0.05. (**b**) Whole cell extracts were subjected to SDS-PAGE and immunoblotting (IB) with anti-p-ERK, anti-ERK, or anti-β-actin antibodies. Similar results were obtained in two separate experiments.

In the final experiments, we examined whether other flavonoids, such as quercetin, luteolin, and apigenin, stimulated LDLR gene expression. Treatment with quercetin for 24 h significantly stimulated LDLR gene expression, whereas treatment with luteolin and apigenin suppressed it (Fig. 7a). Treatment with quercetin, luteolin, and apigenin for 24 h stimulated ERK1/2 phosphorylation in HepG2 cells (Fig. 7b).

## Discussion

Our results demonstrated that the flavonoid kaempferol stimulated Sp1 activity through accelerated phosphorylation on Thr-453 and Thr-739 by ERK1/2 and LDLR gene expression and activity. There is evidence that kaempferol reduces the risk of developing atherosclerosis by its activity against free radical activity and by reduction of LDL oxidation, which leads to suppression of atheroma formation^16^,^17^. Kaempferol treatment in rabbits fed a high-cholesterol diet reduced the formation of plaques and fatty streaks in the aorta with accompanying reduction of the expression of adhesion molecules, such as intercellular adhesion molecule-1 and vascular cell adhesion molecule-1, and reduced serum levels of tumor necrosis factor-α and interleukin-1β^11^. In the present study, we found that kaempferol stimulated the gene expression of LDLR in cultured hepatocytes. It has been reported that dietary kaempferol (150 mg/kg) for 6 weeks reduced serum LDL cholesterol levels in rabbits fed a high-cholesterol diet^11^. Therefore, activation of LDLR in the liver could contribute, at least in part, to the amelioration of atherosclerosis by kaempferol. In the present study, we demonstrated that kaempferol at 50 μM, but not 10 μM, stimulated LDLR gene expression in cultured hepatocytes (Fig. 1b). It has been reported that kaempferol reached a maximum plasma concentration of 1.43 μg/ml (5 μM) after oral administration of 6 mg/kg kaempferol in rats^18^. Considering this, dietary kaempferol at 150 mg/kg in rabbits may result in kaempferol plasma concentrations of >50 μM. Further studies are required to specifically determine the bioavailability of kaempferol and whether dietary kaempferol can stimulate LDLR gene expression in the liver.

We have previously reported that LDLR mRNA was rapidly degraded, with a half-life of approximately 3 h, and stabilized by the bile acid chenodeoxycholic acid^19^. We have also reported that chenodeoxycholic acid treatment activated the ERK pathway, but its stabilization of LDLR mRNA was inhibited in the presence of the MEK inhibitor U0126 in cultured hepatocytes^19^,^20^. Considering that kaempferol stimulated the ERK pathway, kaempferol may stabilize LDLR mRNA, in addition to stimulating LDLR transcription by Sp1, to cause an increase in LDLR mRNA.

It has been known that many cancers contain active mutations in genes encoding for MEK1 or MEK2; consequently, activation of the ERK pathway is deregulated^21^. In many cancers, Sp1 is overexpressed and levels correlate with tumor stage and poor prognosis^22^. In addition, MEK1/2 and Sp1 inhibitors have been used for cancer therapy^21^,^22^. However, several studies have reported that kaempferol inhibited cancer cell growth and induced cancer cell apoptosis, possibly due to reduction of cyclin-dependent kinase 1 levels in breast cancer cells^10^ and reduction of cMyc levels in cisplatin-treated ovarian cancer cells^23^. In addition, kaempferol directly binds to the anti-apoptotic protein RSK2 and inhibits RSK2 activity^24^. Kaempferol also directly binds to and inhibits the activity of Src tyrosine kinase^25^. The effect of kaempferol on the ERK pathway may be dependent on the cell type. Kaempferol downregulates ERK phosphorylation and inhibits VEGF expression through a novel ERK−NFκB−cMyc−p21−VEGF pathway, leading to angioprevention in ovarian cancer cells^26^. Kaempferol-mediated downregulation of ERK phosphorylation in glioma cells decreases the expression of anti-apoptotic proteins survivin and X-linked inhibitor of apoptosis protein, ultimately inducing cell death^27^. On the other hand, Kim *et al.* reported a sustained ERK activation by kaempferol treatment in breast cancer cells^28^. They demonstrated that persistent phosphorylation of ERK1/2 for 24 h after kaempferol treatment was involved in kaempferol-induced apoptosis. In the present study, we found that kaempferol treatment caused early (0.5–3.0 h) and late (24 h) ERK activation in cultured hepatocytes (Fig. 5b), but it did not cause a decrease in cell proliferation (Fig. 2a). Further studies are required to determine how kaempferol affects the ERK pathway in a cell type-specific manner and whether the undulating ERK activation by kaempferol influences the apoptotic process in hepatocytes.

LDLR gene expression has been reported to increase through SREBP activation under sterol-depleted conditions^4^. It has been also reported that LDLR gene promoter was synergistically activated by SREBP and Sp1^6^. Given that kaempferol stimulates Sp1 activity, the kaempferol-mediated stimulation of LDLR gene expression may be enhanced under sterol-depleted conditions. We have previously reported that the alkaloid piperine, a pungent constituent of black pepper and long pepper, induced LDLR expression through proteolytic activation of SREBP^12^. Therefore, it is probable that treatment with both kaempferol and piperine further stimulates LDLR gene expression. Other than Sp1, several transcription factors, such as PPARγ, PPARδ, and AP-1, have been shown to regulate the LDLR gene promoter^29^,^30^,^31^. Additionally, kaempferol regulates several signaling pathways, such as a cAMP–PKA pathway^32^, in addition to the ERK pathway. Clearly, further studies are needed to determine whether multiple transcription factors and signaling pathways are involved in the kaempferol-mediated induction of LDLR gene expression. In addition, the flavonols kaempferol and quercetin stimulated LDLR gene expression that was accompanied by ERK1/2 phosphorylation. In contrast, the flavones luteolin and apigenin suppressed LDLR gene expression and increased ERK1/2 phosphorylation (Fig. 7a,b). Therefore, it was unlikely that the flavonoids that increased ERK1/2 phosphorylation consequently stimulated LDLR gene expression. Further studies are required to determine the molecular mechanisms by which each of the flavonoids affects LDLR gene expression.

In conclusion, we propose that by increasing LDLR gene expression and activity, kaempferol may be a potential agent against atherosclerosis. Further experiments using normal cells, such as primary hepatocytes, or *in vivo* animal studies are required to evaluate the effect of kaempferol on LDLR gene expression in the liver.

## Methods

### Reagents

Kaempferol, fluvastatin, mevalonate, lipoprotein-deficient serum (LPDS), and U0126 were purchased from Sigma. Quercetin, apigenin, and luteolin were purchased from Tokyo chemical industry. Kaempferol, quercetin, apigenin, luteolin, and U0126 were dissolved in dimethylsulfoxide (DMSO), and kaempferol was prepared at the time of use. In this study, the final DMSO concentration in the cultured medium was 0.1%. DiI-labeled LDL was obtained from Molecular Probes.

### Media

Medium A contained Dulbecco’s modified Eagle’s medium (DMEM) supplemented with 100 U/ml penicillin, 100 μg/ml streptomycin, and 10% fetal bovine serum. Medium B contained DMEM supplemented with 100 U/ml penicillin, 100 μg/ml streptomycin, 5% LPDS, 50 μM sodium mevalonate, and 12.5 μM fluvastatin.

### Cell culture

Huh-7/LDLR-Luc cells (i.e., Huh-7 cells that stably express a luciferase reporter and are driven by the LDLR promoter)^12^ were maintained in medium A containing 2 μg/ml blasticidin S. HepG2 cells were maintained in medium A. All cell cultures were incubated at 37 °C with 5% CO_2_ atmosphere.

### Luciferase assays in Huh-7/LDLR-Luc cells

Huh-7/LDLR-Luc cells were plated on 12-well plates at a density of 1 × 10^5^ cells/well before culturing in medium A for 20 h; thereafter, these cells were incubated for 24 h, with and without kaempferol (100 μM). Luciferase activity and protein content in the cell extracts were measured as previously described^33^. Luciferase activity was normalized to total protein content of cell extracts.

### LDL uptake assays

LDL uptake assays were performed as previously described^34^.

### Real-time PCR

Total RNA was extracted from HepG2 cells using Isogen (Wako), according to the manufacturer’s instructions; cDNA was synthesized and amplified from 2 μg of total RNA using a high-capacity cDNA reverse transcription kit (Applied Biosystems). Quantitative real-time PCR (TaqMan probe and SYBR green) analyses were performed using an Applied Biosystems StepOnePlus instrument. Expression was normalized to a glyceraldehyde-3-phosphate dehydrogenase (GAPDH) control. The TaqMan identification numbers for the genes analyzed were as follows: GAPDH, 4352934 and Sp1, Hs00916521. The sequences of the primer sets used were LDLR, 5′-CAGAGGCAGAGCCTGAGTCA-3′ and 5′-CGGGTGTCTCAGGCACTTAA-3′.

### Cell viability assay

Cell viability was determined by MTT assay (Dojindo). HepG2 cells were plated in 96-well plates at a density of 2.0 × 10^4^ cells/well and were cultured with medium A for 24 h. After incubation for another 24 h in the absence or presence of 50 μM, 100 μM, or 150 μM kaempferol, MTT assay was performed according to the manufacturer’s instructions.

### Determination of plasma membrane damage

The plasma membrane damage of HepG2 cells was determined by LDH assay (Takara). HepG2 cells were plated in 96-well plates at a density of 1.5 × 10^3^ cells/well and were cultured with medium A for 24 h. After incubation for another 24 h in the absence or presence of 50 μM, 100 μM, or 150 μM kaempferol, LDH assay was performed according to the manufacturer’s instructions.

### siRNA experiments

Sp1 (sc-29487) siRNA was obtained from Santa Cruz; control siRNA (pGL2 luciferase) was obtained from Bonac. HepG2 cells were transfected with siRNA (40 pmol per 6-well plate) using lipofectamine RNAiMAX (Invitrogen), according to the manufacturer’s instructions.

### Antibodies

Monoclonal anti-LDLR and polyclonal anti-Sp1 antibodies were purchased from Millipore. Polyclonal anti-phospho-ERK1/2 and anti-ERK1/2 antibodies were purchased from Cell Signaling Technology. Polyclonal anti-phospho-Sp1 (Thr-453) antibody was purchased from Abcam and polyclonal anti-phospho-Sp1 (Thr-739) antibody was purchased from LifeSpan BioSciences. Monoclonal anti-β-actin (AC-15) antibody was purchased from Sigma. Peroxidase-conjugated affinity-purified goat anti-rabbit and goat anti-mouse IgGs were purchased from Jackson Immunoresearch Laboratories.

### Immunoblotting

Cells were lysed in RIPA buffer containing 50 mM Tris−HCl (pH 8.0), 150 mM sodium chloride, 0.1% (w/v) sodium dodecyl sulfate (SDS), 0.5% (w/v) deoxycholate, 1% (v/v) Triton X-100, and protease inhibitors. Lysates were subjected to SDS−polyacrylamide gel electrophoresis (PAGE); proteins were transferred onto a PVDF membrane and were probed with the indicated antibodies. Immunoreactive proteins were visualized using Western blotting detection reagents, such as ECL (GE Healthcare) or Immobilon (Millipore). Signals on membranes were detected and quantified using ImageQuant LAS 4000 mini (GE Healthcare).

### Chromatin immunoprecipitation assay

HepG2 cells were grown to confluence in 10-cm dishes with medium A, followed by treatment with a vehicle or 100 μM of kaempferol for 24 h. The cells were then processed for the ChIP assay using a reagent kit (Upstate), according to the manufacturer’s instructions. Immunoprecipitation (IP) was performed using normal rabbit IgG, acetyl-histone H3, or anti-Sp1 antibody. Real-time PCR used the following primers: human LDLR repeat1–3, 5′-GAATCAGAGCTTCACGGGTT-3′ and 5′-CCCACGTCATTTACAGCATTTC-3′; human LDLR distal region, 5′-CAACACAAAAGCAGCCCAGA-3′ and 5′-GCCACACTCGGTGCATAATAAA-3′.

### Statistical analysis

All data were presented as mean ± standard error of the mean (SEM). Statistical analyses were performed using Ekuseru-Toukei Ver.2.0 (Social Survey Research Information). Pairwise comparisons of treatments were made using Student’s *t* test. Multiple comparisons were made using one-way analysis of variance followed by the Bonferroni test. Differences were considered significant at *P* < 0.05.

## Additional Information

**How to cite this article**: Ochiai, A. *et al.* Kaempferol stimulates gene expression of low-density lipoprotein receptor through activation of Sp1 in cultured hepatocytes. *Sci. Rep.* **6**, 24940; doi: 10.1038/srep24940 (2016).
